# Caffeinated chewing gum enhances maximal strength and muscular endurance during bench press and back squat exercises in resistance-trained men

**DOI:** 10.3389/fnut.2025.1540552

**Published:** 2025-01-29

**Authors:** Li Ding, Jue Liu, Yi Yao, Li Guo, Bin Chen, Yinhang Cao, Olivier Girard

**Affiliations:** ^1^School of Athletic Performance, Shanghai University of Sport, Shanghai, China; ^2^Department of Rehabilitation Medicine, Huashan Hospital, Fudan University, Shanghai, China; ^3^School of Exercise and Health, Shanghai University of Sport, Shanghai, China; ^4^Department of Public Physical Education, Fujian Agriculture and Forestry University, Fuzhou, China; ^5^School of Human Sciences (Exercise and Sport Science), The University of Western Australia, Perth, WA, Australia

**Keywords:** caffeine, ergogenic aid, nutritional supplement, one-maximum repetition, repetitions to failure

## Abstract

**Introduction:**

Caffeinated chewing gum, known for its rapid absorption, has not been previously studied for its effects on maximal strength and muscular endurance in resistance exercise. The aim of this study was to determine the efficacy of caffeinated chewing gum on maximal strength and muscular endurance during bench press and back squat exercises.

**Methods:**

In a randomized, double-blind design, 16 resistance-trained males (age: 21.6 ± 2.0 years, height: 176.8 ± 6.1 cm, mass: 79.6 ± 8.8 kg) chewed either caffeinated gum (3 mg/kg) or a placebo gum on two occasions, 1 week apart. After a standardized warm-up, participants chewed the gum for 5 min before performing maximal strength test (one-repetition maximum [1RM]) and muscular endurance test (60% 1RM repetitions to failure) for bench press and back squat exercises. 1RM, number of repetitions, ratings of perceived exertion and pain perception were assessed.

**Results:**

Caffeinated chewing gum significantly improved 1RM in both bench press (105.3 ± 14.5 vs. 100.3 ± 13.4 kg, +5.0% [95% confidence interval (CI): 3.7–6.3%], *p* < 0.01) and back squat (172.3 ± 20.2 vs. 161.9 ± 22.3 kg, +6.8% [95%CI: 4.1–9.5%], *p* < 0.01) exercises with small effect size (Cohen’s *d*: 0.36 [95%CI: 0.09–0.63] and 0.49 [95%CI: 0.22–0.76], respectively), despite similar levels of pain perception and RPE (*p* > 0.05). It also increased the number of repetitions in both bench press (20 ± 5 vs. 17 ± 4, +18.8% [95%CI: 11.5–26.1%], *p* < 0.01) and back squat (37 ± 11 vs. 28 ± 8, +33.3% [95%CI: 23.1–43.4%], *p* < 0.01) exercises with moderate-to-large effect size (Cohen’s *d*: 0.76 [95%CI: 0.48–1.03] and 0.89 [95%CI: 0.60–1.16], respectively), despite similar levels of pain perception and RPE (*p* > 0.05).

**Discussion:**

Caffeinated chewing gum (3 mg/kg) improved both maximal strength and muscular endurance during bench press and back squat exercises in resistance-trained men. This approach offers a practical and time-efficient method to improve training performance while minimizing the risk of side effects.

## 1 Introduction

Caffeine (1,3,7-trimethylxanthine) is a widely used ergogenic supplement among athletes and non-athletes (1). Its performance-enhancing effects have been recognized for over a century (2), with substantial evidence supporting its benefits across aerobic (3, 4), anaerobic (5), and sport-specific exercises (6). Recently, as resistance exercise has gained popularity, research on caffeine’s effects in this domain is also expanding (7–9).

A substantial and growing body of evidence supports the performance-enhancing effects of moderate doses of caffeine (5–6 mg/kg) on maximal strength (one-repetition maximum [1RM]) and muscular endurance (repetition to task failure at 60–70% 1RM) during bench press and/or back squat exercises (10–12), which are common among both recreational and competitive athletes. The primary mechanism is likely the role of caffeine as an adenosine receptor antagonist (8, 13), which may inhibit the negative effects of adenosine on neurotransmission, perceived exertion, pain perception, and arousal (5, 14, 15). In addition, caffeine may increase sodium-potassium pump activity, potentially enhancing excitation contraction coupling (5). Traditionally, caffeine has been consumed in capsules, coffee, or energy drinks approximately 1 h prior to exercise (16–18). However, the slow absorption rate of caffeine from solid or liquid forms through the gastrointestinal tract may lead to fluid overload or gastrointestinal discomfort during intense exercise (19). Therefore, alternative caffeine usage methods that minimize these side effects and facilitate faster absorption are warranted, especially for resistance-trained athletes.

Caffeinated chewing gum offers faster absorption of circulating caffeine compared to capsules, via absorption through the buccal cavity, bypassing first-pass metabolism (5–10 vs. 45–60 min) (20, 21). This method may also minimize the risk of gastrointestinal disorders in athletes (21). Existing studies have shown that gum containing low-to-moderate caffeine doses (2.4–4.5 mg/kg) improves performance in activities such as cycling (21, 22), running (23), and team sports (24). Furthermore, two recent studies found that gum containing relatively low caffeine doses (2.5–3.6 mg/kg) enhances muscular power and explosive performance during resistance exercises (e.g., Romanian deadlift and bench press) (25, 26). However, a review indicated that the effects of caffeine on power may differ from those on maximal strength (1RM) and muscular endurance (repetitions to failure) during resistance exercise (8). Thus, it remains unclear whether gum containing low-dose caffeine (3 mg/kg) also enhances maximal strength and muscular endurance.

The primary purpose of this study was to test the hypothesis that gum containing low dose caffeine (3 mg/kg) would significantly increase maximal strength (1RM) and muscular endurance (number of repetitions) in both bench press and back squat exercises in resistance-trained men.

## 2 Materials and methods

### 2.1 Participants

Sixteen resistance-trained young men were recruited in this study [mean ± standard deviation (SD); age: 21.6 ± 2.0 years; height: 176.8 ± 6.1 cm; body mass: 79.6 ± 8.8 kg; training history: 4.4 ± 1.1 years; 1RM bench press: 99.7 ± 15.6 kg; 1RM back squat: 154.7 ± 22.6 kg]. Participants’ habitual caffeine intake was assessed using the validated Food Frequency Questionnaire (FFQ) and a caffeine intake habit classification table (27, 28), revealing that all participants had naive to mild (0–2.99 mg/kg/day) daily caffeine intake. Inclusion criteria were: (a) young men aged 18–40 years; (b) at least 2 years of resistance training experience, engaging in bench press and back squat exercises at least once weekly; (c) able to perform bench press and back squat exercises with 100 and 125% of body mass, respectively (29). Exclusion criteria included: (a) the presence of any neuromuscular, musculoskeletal, neurological, immunological, or cardio-metabolic disorders; (b) self-reported smoking status; and (c) a self-reported allergy to caffeine (30, 31). This study was approved by the Scientific Research Ethics Committee of Shanghai university of Sport (No. 102772023RT013). During the participant recruitment phase, all participants provided written informed consent.

### 2.2 Experimental design

A randomized, double-blind, placebo-controlled, cross-over design was used to examine the effectiveness of caffeinated chewing gum on maximal strength (1RM) and muscular endurance (number of repetitions at 60% 1RM to failure) during both bench press and back squat exercises. Participants attended the laboratory three times, once for familiarization and preliminary 1RM testing for both exercises and twice for experimental sessions separated by 1 week to ensure full recovery and substance wash-out. All sessions were conducted between 12:00 and 16:00 to avoid potential influences of circadian rhythms (11).

**Figure 1:**
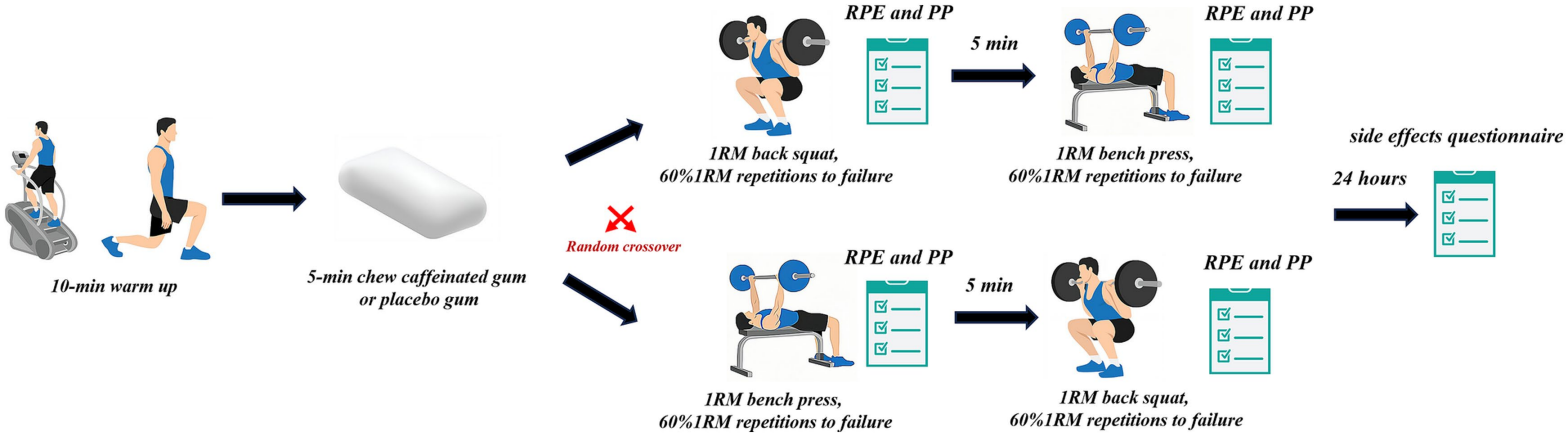
Overview of the experimental protocol.

During each experimental session, participants underwent identical protocols, performing 1RM and 60% 1RM repetitions to failure for both bench press and back squat exercises after chewing either caffeinated gum (3 mg/kg) or placebo gum (Figure 1). Numerous published studies have set the load at 60%1RM (29, 32, 33). An independent researcher used randomization software (Excel Office, Microsoft, Washington, United States) to randomize and counterbalance the intervention order (34). Furthermore, a sensitivity analysis revealed no effects of session order on 1RM (*p* > 0.05; Cohen’s *d*: −0.21 [95%CI: −0.90 to 0.49]) or muscular endurance (*p* > 0.05; Cohen’s *d*: −0.06 [95%CI: −0.76 to 0.63]). Participants were instructed to avoid vigorous exercise and abstain from caffeine intake 24 h before the experiment. All participants were provided with a list of caffeinated items to avoid consumption, such as soda, coffee, chocolate, and energy drinks.

### 2.3 Experimental protocol

During the familiarization session, participants’ height, weight, 1RM bench press, and 1RM back squat were initially assessed. The 1RM was obtained using free-weight equipment (Cybex, Medway, MA, United States) according to the technical guidelines provided by Baechle and Earle (35). This 1RM value was then used to set the load for the 60% 1RM repetitions to failure tests in bench press (59.8 ± 8.4 kg) and back squat (94.6 ± 14.0 kg) exercises in the subsequent experimental sessions. Pilot testing revealed excellent interclass correlation (ICC) for 1RM bench press (ICC = 0.98, 95% confidence interval [CI] = 0.94–0.99) and 1RM back squat (ICC = 0.96, 95% CI = 0.88–0.98) over 2 days in our laboratory. Additionally, participants were instructed to document their diet 24 h prior to this visit and replicate it on the day preceding each experimental session.

Upon arrival for each experimental session, participants engaged in a 10-min general warm-up consisting of 5 min of jogging on a treadmill at a self-selected pace followed by 5 min of self-selected dynamic stretches and joint mobilization exercises. Afterward, participants chewed either placebo or caffeinated chewing gum for 5 min before beginning the 1RM and 60% 1RM repetitions to failure tests for both bench press and back squat exercises. Two experienced spotters monitored and provided feedback on exercise technique to ensure standardization. In addition, to minimize potential interference from exercise sequence and variations in serum caffeine concentration over time on caffeine’s effectiveness (32, 36, 37), the order of the bench press and back squat exercises was randomized and counterbalanced across participants, remaining consistent for each individual during both experimental sessions using randomization software (Excel Office, Microsoft, Washington, United States). Participants were instructed not to wear bench shirts, weightlifting belts, or other supportive garments during the tests (38). After each test, participants were immediately asked to identify the substance they believed they had ingested, and a side effects survey was conducted 24 h later.

#### 2.3.1 Maximal strength (1RM) and muscular endurance (60% 1RM repetitions to failure) tests for bench press exercise

Before determining the 1RM for the bench press exercise, participants performed warm-up sets of 10 and 5 repetitions using 50 and 75% of their 1RM measured in familiarization session, respectively (39). After a 5-min rest, participants initiated their first attempt at 95% 1RM established during the familiarization session (40). Following each successful attempt, the load was increased by 2.5–5 kg until the participant failed to complete a full range of motion (11, 38). If an attempt failed, the weight was decreased by 2.5–5 kg until the 1RM was determined (11, 38). Verbal encouragement was provided during each attempt. Thus, the 1RM was determined within 3–5 attempts, with a 3-min rest between each attempt (41). To ensure safety, all 1RM attempts were conducted with safety pins and two experienced spotters present.

After a 5-min passive rest, participants proceeded to the muscular endurance test, which involved performing bench press repetitions to failure at 60% 1RM, following a 2/0/X/0 cadence (2 s for the eccentric phase, no pause during the transition phase, maximum tempo during the concentric phase, and no pause at the end of the movement) (42). The cadence was controlled by a metronome set to 30 beats/min (10). The muscular endurance test was terminated when momentary concentric failure occurred (38, 42). The total number of repetitions performed to failure was recorded as an index of muscular endurance performance (43).

#### 2.3.2 Maximal strength (1RM) and muscular endurance (60% 1RM repetitions to failure) tests for back squat exercise

After a 5-min passive rest, participants completed warm-up sets of 10 and 5 repetitions of the back squat exercise using 50 and 75% of their pre-measured 1RM, respectively. Following a 3-min rest, they measured their 1RM in the back squat exercise using the same protocol as for the bench press exercise. Participants positioned their feet slightly wider than shoulder width, adhering to recommendations of Baechle and Earle (35). A successful repetition required completing the back squat exercise until the hips and knees reached the same level in the concentric phase (39). Therefore, a bungee cord was positioned at the parallel back squat depth, and participants had to touch the cord with their buttocks before ascending (44). After determining the 1RM, participants rested for 5 min and then performed a 60% 1RM repetitions-to-failure test for the back squat exercise, following the same procedure and standards as the bench press. The total number of repetitions performed to failure was also recorded as an index of muscular endurance performance.

### 2.4 Ratings of perceived exertion and pain perception

Ratings of perceived exertion (RPE) and pain perception were assessed using the validated 6–20 point RPE scale and the 0–10 point pain perception scale (45, 46), respectively. These assessments were conducted within 5 s of a successful 1RM attempt and upon completion of the muscular endurance test. Prior to each trial, participants received instructions on the proper use of these scales.

### 2.5 Administration of caffeinated and placebo chewing gum

In each trial, participants chewed the gum for 5 min before expectorating it into a container. It has been previously reported that 85% of the dose is released within this time frame (20). Chewing was timed using a handheld stopwatch. Each gum contained 3 mg/kg of caffeine from a commercially available chewing gum (Military Energy gum; Market Right Inc., Plano, IL, United States), as this dosage is considered the threshold for ergogenic effects in resistance exercise (47, 48). The placebo was a commercially available non-caffeinated chewing gum (Spearmint Extra professional; Wrigley’s, Chicago, IL, United States), similar in taste, shape and size. All gum samples were weighted using a high-precision electronic digital scale, wrapped in uniform foil, and administered by a designated experimenter.

### 2.6 Assessment of blinding and side effects

At the end of each experimental session (20), the effectiveness of the blind procedure was assessed by asking participants: “*What substance do you think you ingested?*.” Response options included: (a) caffeinated chewing gum; (b) placebo chewing gum; and (c) I do not know (49). Additionally, 24 h after completing the test, participants filled out a side effects questionnaire (QUEST), a nine-item dichotomous scale assessing side effects associated with caffeine consumption (38).

### 2.7 Statistical analysis

Data were reported as means ± standard deviation (SD). The normality of the sample data was assessed using the Shapiro–Wilk test. A paired sample *t*-test was used to investigate differences in performance outcomes (1RM, number of repetitions) and perceptual responses (RPE and pain perception) between conditions. Effect sizes were reported using Cohen’s *d* (trivial: 0 ≤ *d* < 0.2; small: 0.2 ≤ *d* < 0.5; moderate: 0.5 ≤ *d* < 0.8; large: *d* ≥ 0.8) (50). Statistical significance was set at *p* < 0.05. In addition, the effectiveness of blinding was assessed using Bang’s Blinding Index (BBI), with a value of −1.0 indicating complete opposite guessing and a value of 1.0 indicating a complete lack of blinding (51). All data analyzes were carried out using SPSS (version 22.0; SPSS Inc., Chicago, IL, United States).

## 3 Results

### 3.1 Maximal strength

**Figure 2:**
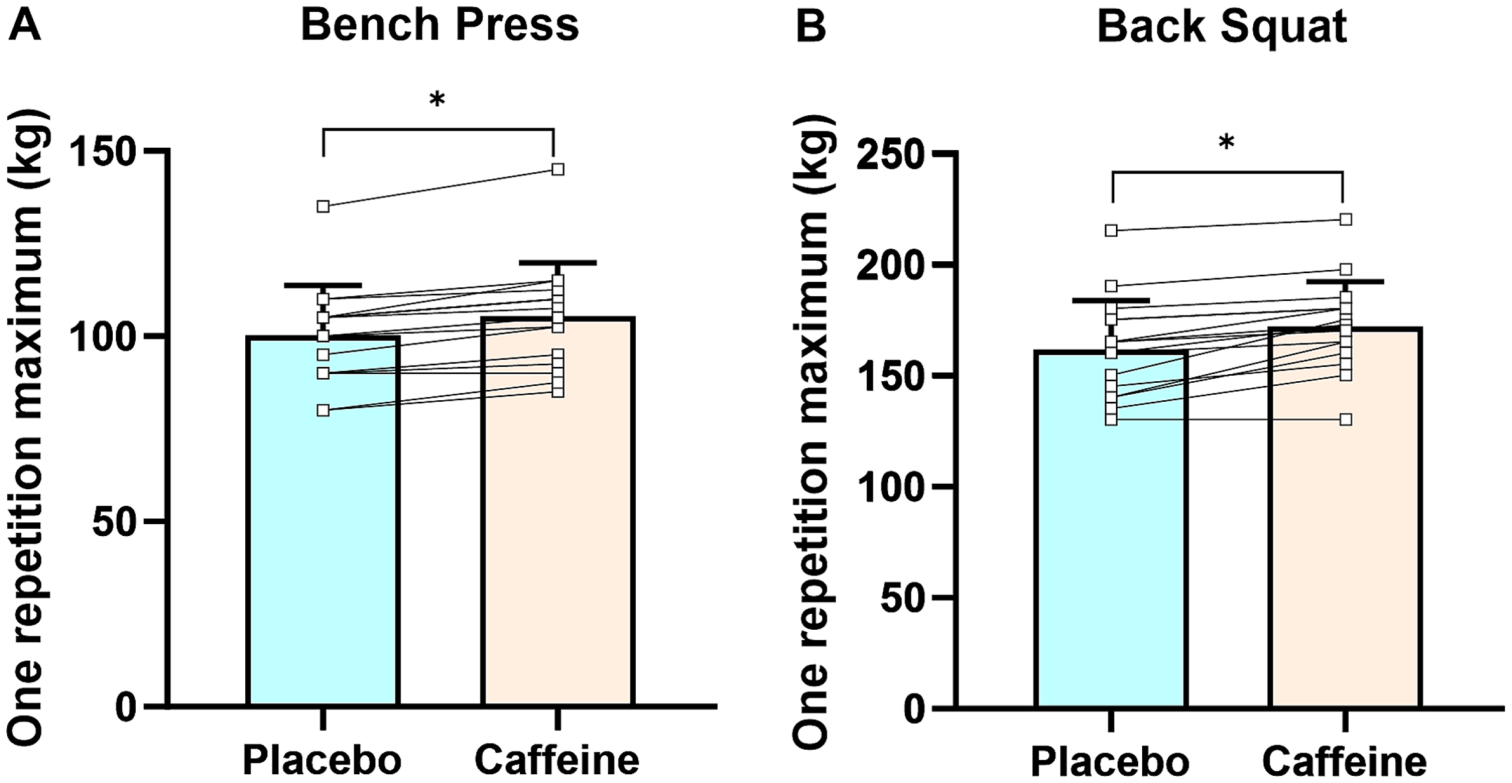
The one-repetition maximum in bench press **(A)** and back squat **(B)** exercises. Error bars represent standard deviation. *p < 0.05 caffeine vs. placebo trials.

Caffeinated chewing gum significantly increased 1RM for both bench press (105.3 ± 14.5 vs. 100.3 ± 13.4 kg, +5.0% [95% confidence interval (CI): 3.7–6.3%], *p* < 0.01) and back squat (172.3 ± 20.2 vs. 161.9 ± 22.3 kg, +6.8 [95%CI: 4.1–9.5%], *p* < 0.01) exercises with small effect size (Cohen’s *d*: 0.36 [95%CI: 0.09–0.63] and 0.49 [95%CI: 0.22–0.76], respectively), compared to placebo chewing gum (Figures 2A,B).

### 3.2 Muscular endurance

**Figure 3:**
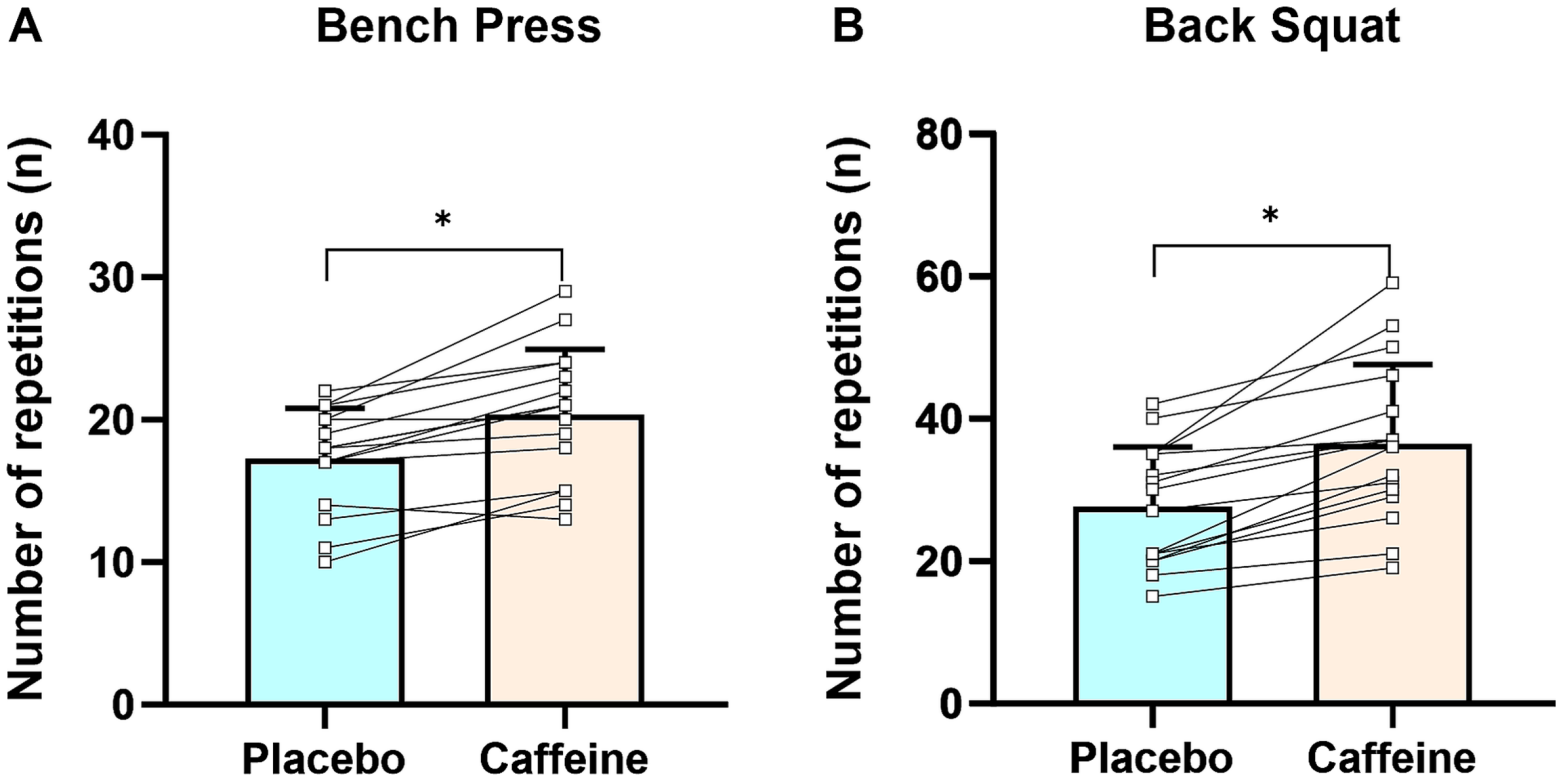
The number of repetitions in bench press **(A)** and back squat **(B)**. Error bars represent standard deviation. *p < 0.05 caffeine vs. placebo trials.

Caffeinated chewing gum resulted in improvement in number of repetitions for both bench press (20 ± 5 vs. 17 ± 4, +18.8% [95%CI: 11.5–26.1%], *p* < 0.01) and back squat (37 ± 11 vs. 28 ± 8, +33.3% [95%CI: 23.1–43.4%], *p* < 0.01) exercises with moderate-to-large effect size (Cohen’s *d*: 0.76 [95%CI: 0.48–1.03] and 0.89 [95%CI: 0.60–1.16], respectively), compared to placebo chewing gum (Figures 3A,B).

### 3.3 Perceptual responses

**Figure 4:**
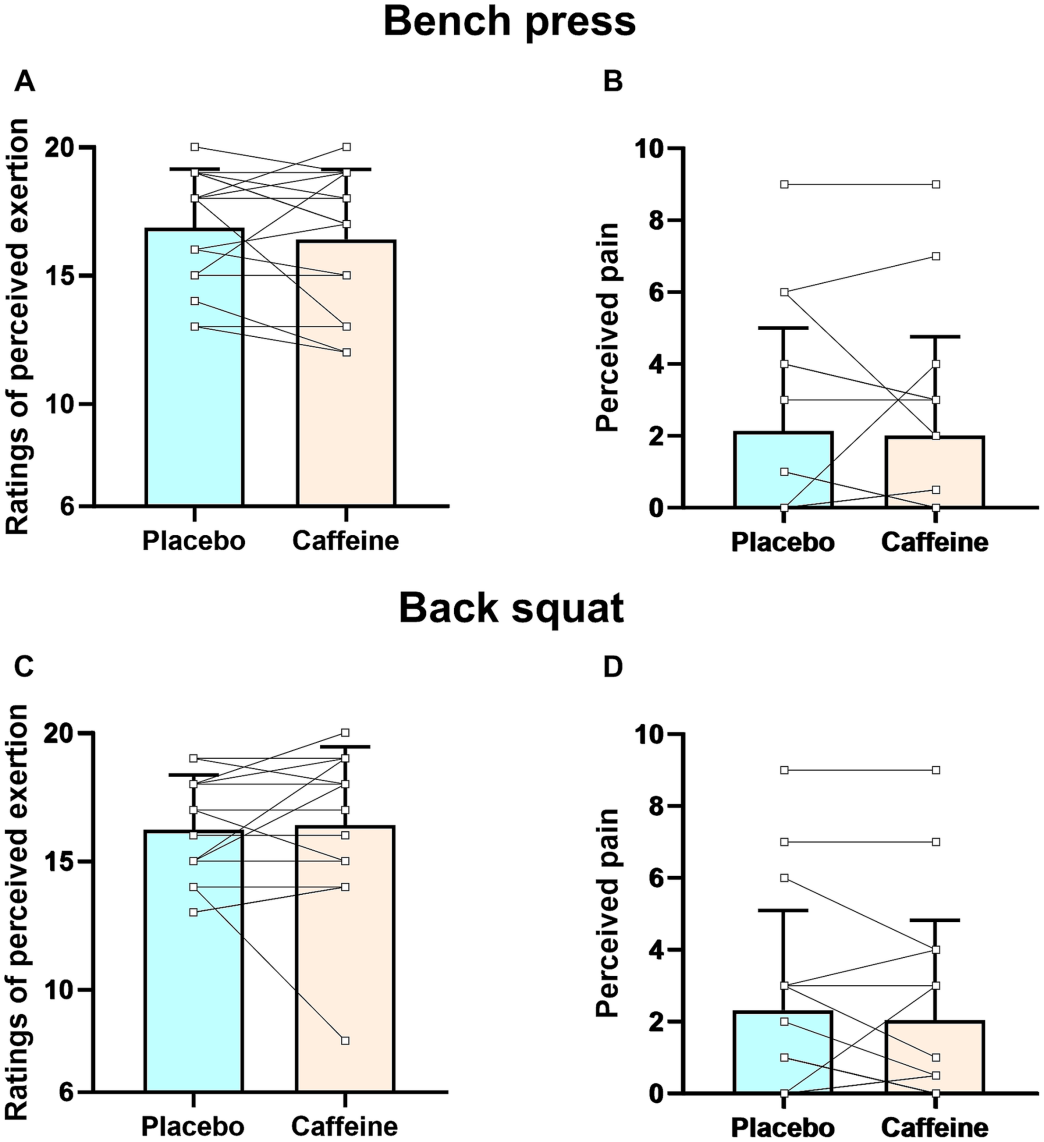
Ratings of perceived exertion and perceived pain during muscular strength test for bench press **(A,B)** and back squat **(C,D)** exercises. Error bars represent standard deviation.

**Figure 5:**
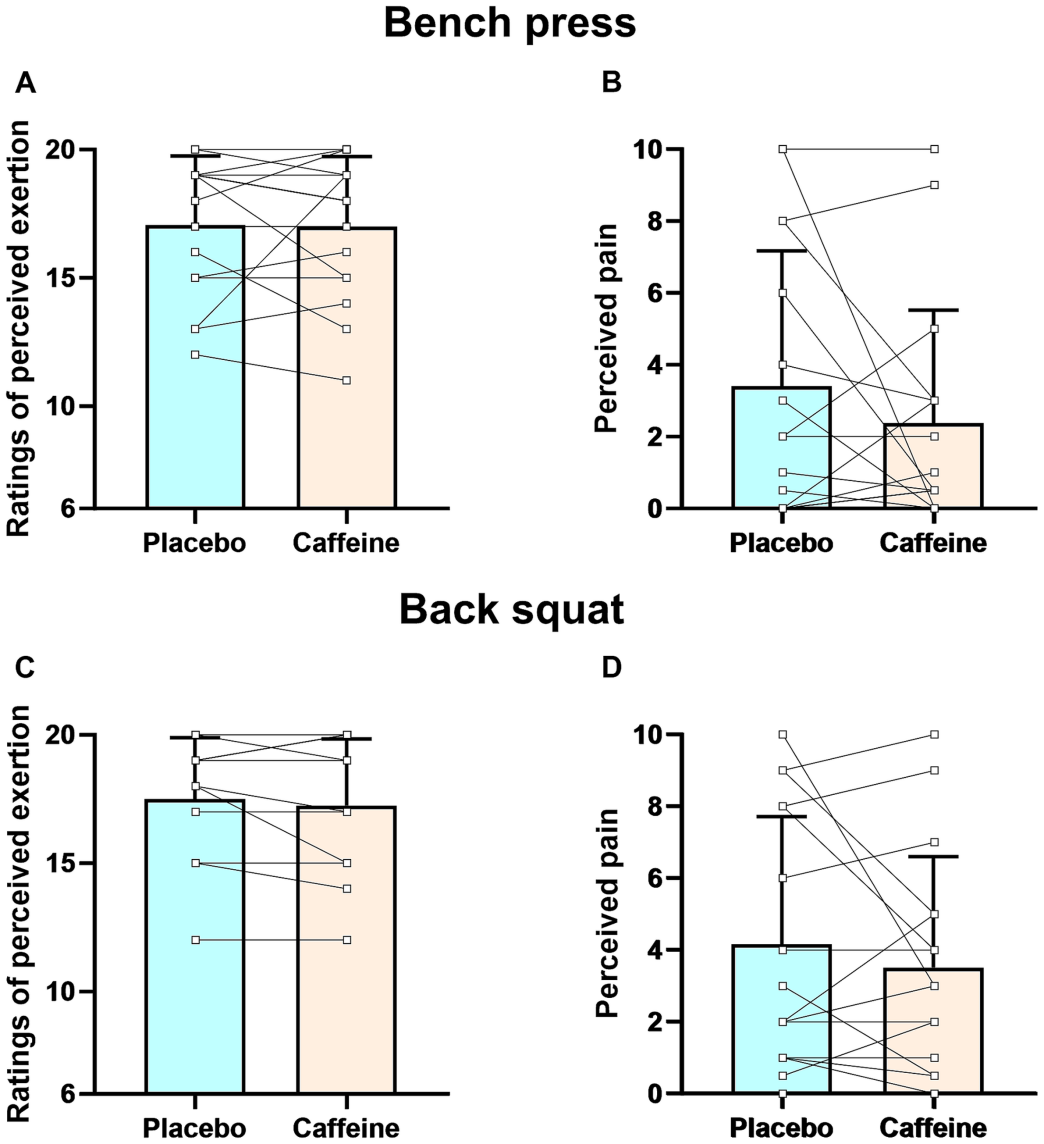
Ratings of perceived exertion and perceived pain during muscular endurance test for bench press **(A,B)** and back squat **(C,D)** exercises. Error bars represent standard deviation.

RPE and pain perception did not differ between conditions in either bench press or back squat exercises (all *p* > 0.05) (Figures 4, 5).

### 3.4 Assessment of blinding and side effects

The statistical results indicated that blinding was generally effective in both the caffeine group (BBI: 0.31 [95%CI: −0.06 to 0.69]) and placebo group (BBI: 0.18 [95% CI: −0.20 to 0.58]). Additionally, 37.5% of participants reported side effects, including increased urine output, gastrointestinal problems, anxiety or nervousness, tachycardia and heart palpitations, and insomnia (Table 1).

**Table 1:** The number (frequency) of participants who reported side effects 24 h later, out of 16 participants.

Variables	Placebo	Caffeine
Increased urine output	2 (12.5%)	7 (37.5%)
Muscle soreness	0 (0%)	0 (0%)
Headache	0 (0%)	0 (0%)
Tachycardia and heart palpitations	0 (0%)	1 (6.25%)
Increased vigor/activeness	0 (0%)	0 (0%)
Anxiety or nervousness	1 (6.25%)	2 (12.5%)
Gastrointestinal problems	1 (6.25%)	2 (12.5%)
Insomnia	0 (0%)	1 (6.25%)
Perception of performance improvement	0 (0%)	0 (0%)

Both absolute (number of participants) and relative (frequency) data are presented.

## 4 Discussion

The major finding of this study is that chewing a low dose of caffeinated gum (3 mg/kg) for 5 min significantly increases 1RM and the number of repetitions to failure during both bench press and back squat resistance exercises, without affecting RPE or pain perception. These results suggest that caffeinated chewing gum is an effective ergogenic aid for enhancing both upper and lower limb maximal strength and muscular endurance performance in resistance-trained men.

### 4.1 Effect of caffeinated chewing gum on maximal strength

Our results revealed that chewing 3 mg/kg of caffeinated gum for 5 min significantly increased 1RM for both bench press and back squat exercises (Figure 2). This is consistent with the meta-analysis by Grgic et al. (7), which demonstrated that caffeine intake (3–7 mg/kg) in traditional forms (e.g., capsules and liquids) significantly improved upper and lower body maximal strength in men. Furthermore, the effect sizes (Cohen’s *d*) observed in our study for caffeinated chewing gum (bench press: 0.36 [95%CI: 0.09–0.63], back squat: 0.49 [95%CI: 0.22–0.76]) are comparable to recent research on caffeine intake (3–6 mg/kg) in capsule form, which improved 1RM for bench press (Cohen’s *d*: 0.17–0.40) (11, 42, 52) and back squat (Cohen’s *d:* 0.20–0.30) (29, 33) exercises in resistance-trained participants. Additionally, our study builds on previous research showing that caffeinated chewing gum significantly increases muscular power (movement velocity and power output) (25, 26), and provides new evidence of its potential to enhance maximal strength, further supporting its role as an effective ergogenic aid for resistance exercise. Mechanistically, caffeinated chewing gum allows caffeine to be absorbed into the bloodstream either through the gastrointestinal tract via swallowed saliva and/or directly through the extensive vascularization of the buccal mucosa, enabling it to compete for adenosine binding sites in the central nervous system (48). This competition leads to increased hormone secretion (e.g., catecholamines and acetylcholine) (53), enhanced neuromuscular recruitment and faster firing rates (15), ultimately enhancing maximal strength. In addition, caffeine entering systemic circulation can also directly act on skeletal muscle (20), promoting the release and reuptake of calcium ions from the sarcoplasmic reticulum, ultimately increasing cross-bridge formation and enhancing maximal strength (5). Furthermore, the potential mechanism of bitter taste stimulation induced by caffeinated chewing gum should not be overlooked. Specifically, caffeine may bind to bitter taste receptors in the mouth, stimulating brain regions responsible for motor control and emotional processing, thereby enhancing central arousal and improving resistance exercise performance (54, 55).

Substantial evidence supports caffeine’s ergogenic effects on upper and lower body maximal strength (11, 33, 42), but some studies report conflicting results (21, 56, 57). For example, previous research found that caffeine intake in tablet form (400 mg and 201 mg) did not improve 1RM bench press and 1RM back squat in untrained individuals (56, 57). This discrepancy may be attributed to the participants’ training status. Trained individuals often exhibit greater mental discipline, enabling them to harness more pronounced ergogenic effects from caffeine compared to untrained individuals (58). Alternatively, trained individuals may possess a higher density of adenosine receptors, making them more responsive to caffeine, which could lead to greater benefits (59, 60). However, our study focused solely on resistance-trained individuals, leaving the effects of caffeinated chewing gum on 1RM in untrained individuals uncertain, which warrants further investigation. Additionally, Wilk et al. (38) reported no significant enhancement in 1RM for bench press following two doses of caffeine (9 and 11 mg/kg) in trained athletes. This might be attributed to the high caffeine doses, resulting in severe side effects in 90% of cases (38), ultimately reducing the ergogenic effects of caffeine (21).

### 4.2 Effect of caffeinated chewing gum on muscular endurance

Our study found that chewing 3 mg/kg caffeinated gum significantly increased number of repetitions for both bench press and back squat at 60% 1RM (Figure 3). This aligns with Ferreira et al.’s meta-analysis, which found that caffeine intake (3–11 mg/kg) in traditional forms (e.g., capsules and liquids) significantly increased repetitions for bench press at loads ranging from 30 to 80% 1RM (61). The effect sizes (Cohen’s *d*) in our study (bench press: 0.76 [95%CI: 0.48–1.03], back squat: 0.89 [95%CI: 0.60–1.16]) were consistent with those observed in other research on caffeine ingestion (4–6 mg/kg) in capsule form, which enhanced the number of repetitions for bench press (Cohen’s *d*: 0.33–0.58) (12, 33) and back squat (Cohen’s *d*: 0.35–0.90) (11, 12, 33) at 60–70% 1RM in resistance-trained individual. This suggests that, compared to capsules, caffeinated chewing gum can produce similar effects at relatively low dosage (3 mg/kg vs. 4–6 mg/kg). Fatigue from a decline in neural drive significantly influences muscular endurance during resistance exercise (31, 62). The improved muscular endurance observed here is likely primarily due to the ‘anti-fatigue’ effects of caffeine chewing gum (63). Caffeinated gum can reduce RPE and pain perception (39), thereby deteriorating central inhibitory motor control (64), slowing the decline in neural drive, and improve muscular endurance (60). Grgic et al. (39) suggested that caffeine ingestion can improve power output at a given level of pain perception and RPE. Our results support this, showing that caffeinated chewing gum increased the number of repetitions for both bench press and back squat compared to placebo, despite similar levels of pain perception and RPE (Figure 5).

Astorino et al. (32) indicated that ingesting a moderate dose of caffeine (6 mg/kg) in capsule form did not improve the number of the bench press repetitions in resistance-trained individuals. This result contrasts with our results and those of comparable research (11, 12, 33), which may be explained by the inclusion of higher caffeine consumers (600 mg/day) in their study, as well as insufficient rest between the 1RM and the muscular endurance tests. Habitual caffeine intake (≥3 mg/kg/day) can reduce its ergogenic effect on exercise performance by increasing the body’s tolerance to caffeine (3, 65), possibly reducing its ergogenic effect on muscular endurance during bench press. Furthermore, in the study by Astorino et al. (32) the muscular endurance test was conducted immediately after the 1RM test, with no sufficient rest period. This may have exacerbated fatigue, potentially diminishing the effects of caffeine (29, 40). Additionally, in the study by Astorino et al. (32), 1RM was measured after participants ingested substances during each session. Since our study demonstrated that caffeine significantly increased 1RM (Figure 2), these contradictory results may be attributed to differences in the absolute load at 60%1RM. However, further research is needed to confirm this.

### 4.3 Side effects of caffeinated chewing gum

Paton et al. (21) indicated that caffeinated chewing gum may cause fewer side effects compared to capsules. Our study supports this, showing a lower incidence of side effects (i.e., muscle soreness: 0% vs. 24%; insomnia: 6.3% vs. 34%) compared to a systematic review involving 421 participants that examined the side effects of low-dose caffeine (≤3 mg/kg) intake capsules, liquids, and energy drinks (66). Another study on the effects of the same caffeine dose (3 mg/kg) in capsule form on resistance performance in resistance-trained men reported a higher incidence of side effects, particularly gastrointestinal issues (6.3% vs. 61.5%). This difference may be attributed to the absorption of caffeine from chewing gum primarily occurring through the buccal cavity’s extensive vascularization, bypassing intestinal and hepatic first-pass metabolism, which may reduce gastrointestinal side effects (20, 21). Future research should directly compare the side effects of these two forms of caffeine intake to provide more definitive evidence.

### 4.4 Limitations and additional considerations

Our study has certain limitations. Firstly, we did not measure brain activity, muscle electrical signals, or neurotransmitters concentrations, which could provide a more comprehensive understanding of caffeine’s effects on maximal strength and muscular endurance. Secondly, although caffeinated chewing gum seems to have a lower incidence of muscle soreness and insomnia compared to traditional caffeine intake forms (e.g., capsule, tablet, and liquid), the underlying mechanisms remain unclear and warrant investigation. Thirdly, *a priori* power analysis (G*Power version 3.1, Universität Düsseldorf, Düsseldorf, Germany) indicated a minimum sample size of 28, based on an α-level of 0.05, a 1-β error probability of 0.8, a correlation of 0.90, and an effect size (*f*) of 0.125 for the back squat, as reported by Norum et al. (33). This suggests that the sample sizes in our study (*n* = 16) and others (*n* = 15–19) (26, 29, 67) may be underpowered. However, given the effect sizes (Cohen’s *d*: 0.36–0.89) and all *p*-values < 0.01 for 1RM and the number of repetitions in our study, increasing the sample size is unlikely to substantially alter our results. Fourthly, our study focused exclusively on the effects of a low dose caffeinated chewing gum (3 mg/kg) on muscular strength and endurance in resistance-trained men. Due to difference in genotypes, habitual caffeine intake, and training status (60), there is large individual variability in the ergogenic effects of caffeine. Future research should seek to establish an “optimal” dosage tailored to different populations, as well as to explore the mechanisms underlying the enhanced maximal strength and muscular endurance associated with caffeinated chewing gum.

### 4.5 Practical application

Our study indicates that chewing caffeinated gum (3 mg/kg) for 5 min before resistance exercise can enhance both maximal strength and muscular endurance during bench press and back squat exercises. Considering that these exercises are widely performed by both recreational and competitive athletes, many of whom train in the morning, we recommend caffeinated chewing gum as a practical and time-effective means to enhance training performance and reduce the risk of side effects.

## 5 Conclusion

Caffeinated chewing gum (3 mg/kg) improved both maximal strength and muscular endurance during bench press and back squat exercises in resistance-trained men. This approach offers a practical and time-efficient method to improve training performance while minimizing the risk of side effects.

## Data Availability

The raw data supporting the conclusions of this article will be made available by the authors, without undue reservation.
